# Rural Versus Urban Mothers' Microbiome Difference and Its Effect on Neonates: A Systematic Review

**DOI:** 10.7759/cureus.55607

**Published:** 2024-03-05

**Authors:** Soumya Anumula, Krishna Nalla, Paramesh Pandala, Rakesh Kotha, Neelam Harsha

**Affiliations:** 1 Pediatrics, Government Medical College Vikarabad, Vikarabad, IND; 2 Community Medicine, Government Medical College Jangaon, Jangaon, IND; 3 Pediatrics, Government Medical College Jangaon, Jangaon, IND; 4 Neonatology, Osmania Medical College, Hyderabad, IND; 5 Neonatology, Niloufer Hospital, Hyderabad, IND

**Keywords:** obesity, neonate, urban mothers, rural mothers, microbiota, microbiome

## Abstract

The growth and development of microorganisms are stimulated by external stimuli. Urbanization has changed the macroenvironment and individual microenvironmental factors such as smoking, alcohol, and diet, which can alter the microbiota and influence disease in the mother and child. However, the microbiome difference between rural and urban mothers and its effect on neonates have received little attention, as per sources; we have not found any systematic review. This review determined the microbiome difference between rural and urban mothers and its effect on neonates. Five studies selected based on inclusion/exclusion criteria were retrieved from PubMed, Scopus, and Embase databases, and evidence-based comparisons were made to establish the microbiome difference in rural and urban mothers and its effect on neonates. The study findings indicate that microbiome development in newborns is hindered by urbanization. Infants born to urban mothers have reduced microbial diversity, thereby having decreased protective immunity.

## Introduction and background

The human microbiome comprises organisms such as bacteria and viruses that inhabit and interact with the human body. Steady interactions between these organisms and the human body are essential for maintaining one’s health and well-being [1]. An alteration in the composition of the human microbiome can cause unsteady interactions, leading to life-threatening diseases. For example, a disruption of the gut microbiota can lead to sepsis. A disruption of the microbiota is more harmful in the early years of life, given that the microbiota is crucial for a child's rapid growth and development. About one million microbial-influenced new neural connections are formed every second in the first one thousand days of life [1]. Environmental factors and lifestyle have been proven to alter the human microbiome composition, making it difficult for neonates’ bodies to establish the required neural connections as well as symbiotic interactions critical for their health and well-being. During pregnancy, an expectant mother's microbiota affects the fetus's development [2]. The environment in which the expectant mother lives and the place of giving birth influence the neonatal microbiota by controlling the innate immune system of the offspring through processes such as metabolism and digestion. Changes in the maternal microbiota resulting from environmental factors may result in negative pregnancy outcomes that compromise the health and welfare of the unborn neonate [3]. Rural and urban environments have been hypothesized to cause microbiome differences in rural and urban mothers. However, the association of rural and urban environments with microbiome differences in rural and urban mothers and the effect it presents on neonates have received little attention. Emerging discourses argue that urbanization may be responsible for microbiome differences, making neonates more susceptible to life-threatening illnesses [4].

Urban mothers may differ from rural mothers regarding the quality of medical interventions, diets, and sanitation [4]. Medical interventions such as cesarean sections, processed foods or low-fiber foods, sewage disposal, and antibiotic use are associated with urban environments. These differences could impact the neonatal microbiota differently, leading to different health outcomes for newborns [5]. It has been hypothesized that diets, access to better sanitation, and antibiotic exposure lead to the loss of microbial taxa. Emerging discourse indicates that combining rural and urban environmental factors can increase newborns' immune systems [6]. This study aimed to show the microbiome difference between rural and urban mothers and how it affects neonates.

## Review

Method

Search Strategy

Literature on the microbiome difference between rural and urban mothers and its effect on neonates was searched to collect evidence. The research question and topic were identified to provide a guide on the study characteristics and areas of interest. The research question was formed within the bounds of the research topic. Words used in the research topic were searched the same way they appeared or using related terms to make the search more specific. 

**Figure 1:**
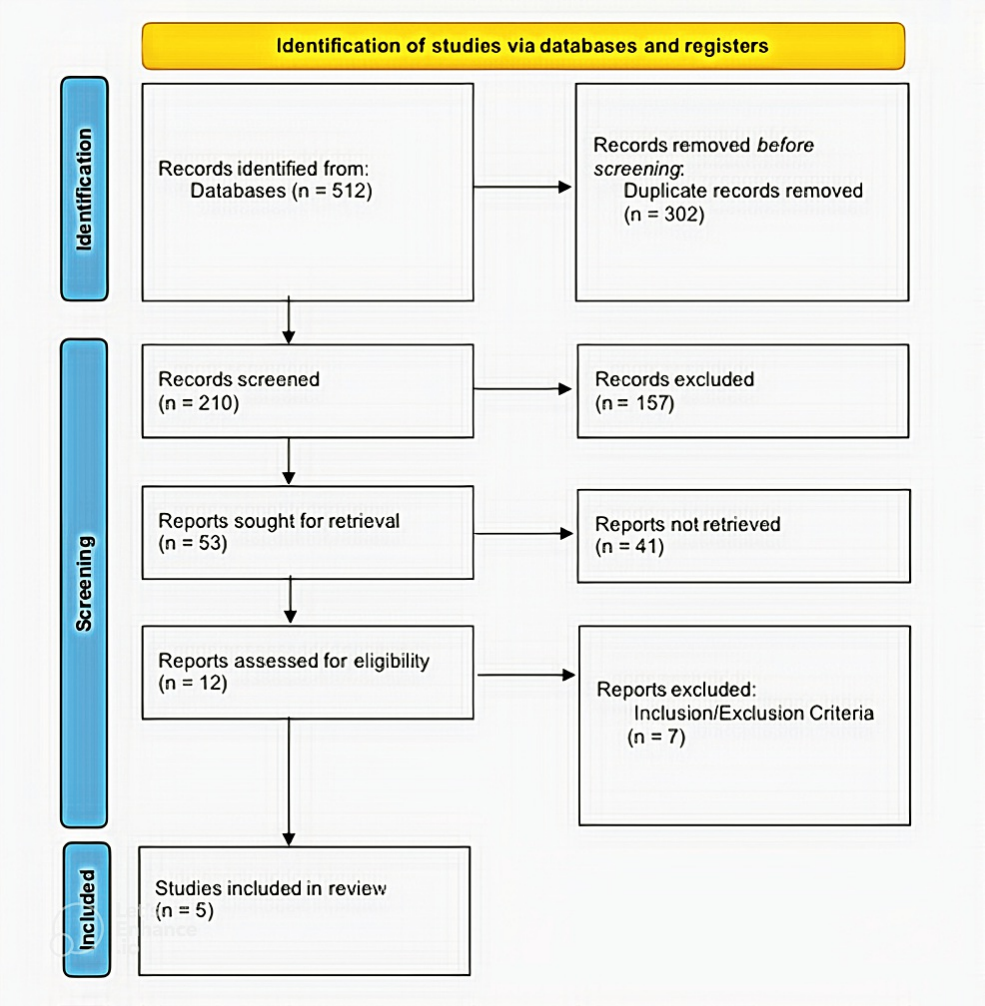
PRISMA flow diagram PRISMA: Preferred Reporting Items for Systematic Reviews and Meta-Analyses

Using research topic terms, we identified MeSH words. Key concepts and words of the research topic were identified, organized, and combined to be used in searching studies on microbiome differences between rural and urban mothers and their effect on neonates. Elements and concepts of the research topic were used interchangeably to enhance the outcome of the search exercise. PubMed, Scopus, and Embase were used as sources for the studies addressing microbiome in rural and urban mothers and its effects on neonates. Keywords of the research topic were combined using Boolean operators and keyed into the search boxes of these databases, retrieving many reviews screened to identify those similar to the research topic (Table 1). The literature selection entailed screening titles and abstracts and reviewing full texts to find studies that address microbiome in rural and urban mothers and its effects on neonates. Selected studies were eliminated based on our inclusion and exclusion criteria (Table 2). We registered this review under PROSPERO with ID 511107. We used the PRISMA flowchart to summarize our screening process (Figure 1). 

**Table 1:** Search strategy

Search strategy	
#1 Search	Rural mothers vs. urban mothers’ microbiome difference and effect on neonate [Mesh terms]
#2 Keywords search	The Urban Microbiome and Rural Microbiome and their Effect on Neonates [Title/Abstract]
#3 Keyword search	Rural mothers and urban mothers microbiome difference and how it affects neonates [Truncation]
#4 Keyword search	Urbanization and mother-infant microbiota [Truncation]
#5 Keyword search	Urban maternal microbiome on neonates [Truncation]
# 6 Keyword search	Urban maternal microbiome in delaying neonatal maturation [Alternative combinations, alternative terms]

**Table 2:** Inclusion and exclusion criteria

Inclusion criteria	Exclusion criteria
Cohort Studies	Articles with abstracts only
At least one randomized controlled trial (RCT) study, observational study, longitudinal study, prospective observational study, prospective cohort study, and experimental study	Editorial commentary articles
Studies focusing on rural mothers' and urban mothers' microbiome differences and their effect on neonates.	Conference papers

Quality assessment

The quality of the observational studies was based on the Newcastle-Ottawa scale (Table 3). Each study was rated using a “star” system for eight items grouped into selection of participants, comparison of study groups, and the outcome of interest domains. All the studies included in this research earned three stars in terms of selection, two stars for comparability, and three stars for outcome of interest. The selected studies were representative of the cohorts, particularly groups susceptible to microbiome changes, and were drawn from the same community as the cohort. The quality assessment of one experimental study, mainly done by the RoB-2 tool, comes under the low risk of bias category.

**Table 3:** Quality ratings of the selected articles based on the Newcastle-Ottawa scale

S.No	Study	Selection	Comparability	Outcome
1	Morandini et al., (2023) [7]	***	**	***
2	Selma-Royo et al. (2020) [8]	***	**	***
3	Combellick et al. (2018) [9]	***	**	***
4	Kortman et al. (2023) [10]	***	**	***

Results 

A first attempt was made to retrieve 512 articles. Upon review, 302 duplicate studies were dropped from the pool. The remaining 210 studies were subjected to further review. Based on the relevance criterion, 157 articles were disregarded for being irrelevant. The remaining 53 articles were filtered based on how they directly addressed the research topic. A careful examination rendered 41 studies inadmissible for this study. The remaining 12 studies were subjected to inclusion/exclusion criteria, leaving five studies approved to be used for this systematic review (Tables 4, 5).

**Table 4:** Summary of the studies

Authors	Objective/Aim	Study Country/Area	Outcome
Morandini et al., (2023) [7]	To establish urbanization is associated with detrimental microbiome diversity as well as delayed maturation in infants.	Gut microbiomes of mothers in rural and urban Senegal were compared to establish their association with neonatal microbiomes.	Urban mothers have different microbiome compositions from rural mothers, showing a delayed microbiome maturation.
Vaidya et al., (2017) [11]	To understand the relationship between rural and urban lifestyles with milk microbiota.	This study was conducted among 15 rural mothers and 15 urban mothers in India.	Urban and rural lifestyles of mothers account for different microbial diversity and richness. Urban mothers reported low microbial diversity. and richness.
Selma-Royo et al. (2020) [8]	To understand how prenatal environment shapes microbiota colonization and infant growth.	Biological samples of infants from Hospital La Fe, Hospital Clinic, and Parc de Salut from Spain were used to conduct this study.	Urban mothers have low microbial diversity and richness. Urban mothers present delayed colonization of Bacteroides.
Combellick et al. (2018) [9]	To understand microbiome differences of neonates born in rural residences and in hospital.	The study was conducted among infants delivered in rural and urban/hospital areas of New York, USA.	Neonates born in hospital had lower Bacteroides.
Kortman et al., (2023) [10]	To investigate how mothers’ breast milk composition and infant microbiota differ in rural and urban mothers.	This study was conducted in five distinct rural and urban areas in Vietnam.	The microbiota of urban mothers may slow infant microbial development.

**Table 5:** Type of studies NOS: Newcastle-Ottawa scale, RoB 2 tool: Risk of Bias 2 tool

Studies	Type of Study	Rationale	Scale Used
Combellick et al., (2018) [9]	Prospective cohort study	Quality data on primary, exposure, and confounding variables	NOS
Kortman et al., (2023) [10]	Observational Study	High data validity	NOS
Morandini et al., (2023) [7]	Longitudinal Study	Tracks incidences over time	NOS
Selma-Royo et al. (2020) [8]	Prospective cohort study	Quality data on primary, exposure, and confounding variables	NOS
Vaidya et al., (2017) [11]	Experimental	Mechanistic understanding of the variables	RoB-2 tool

Description of individual studies

Morandini et al. (2023) investigated the effect of urbanization on microbiome maturation and diversity [7]. The study compared the gut microbiomes of mothers and newborns from Senegal’s rural and urban areas, deducing the microbiome difference and its effect on neonates. The study was based on the presumption that alterations of the microbiome arising from post-industrial lifestyles associated with urbanization lead to or aggravate diseases that undermine neonates' health and well-being. They collected the samples from urban and rural areas at six months and one year. At six months, there were no differences in microbiota between rural and urban groups.

Over a year, the most common phyla were Firmicutes (73%), Actinobacteriota (10%), Bacteroidota (9%), and Proteobacteria (4%). Rural mothers had a lower abundance of Firmicutes than urban individuals, but no other significant differences were found (p = 0.036, 0.607, 0.503, and 0.221). Delayed maturation of the gut microbiome was observed in urban infants, with lower alpha diversity and higher beta diversity from mothers compared to their rural counterparts. This phenomenon is linked to weakened immune defenses or a higher occurrence of allergies. Moreover, medical examinations revealed that one-year-old urban infants showed a higher prevalence of diseases such as infections, respiratory issues, and dermatological conditions (16/27) compared to rural infants (6/27) with an odds ratio of 4.93 and p-value 0.012. Morandini et al. (2023) found that urban infants showed delayed microbiome maturation compared to rural infants, making them more susceptible to infectious diseases [7]. 

Vaidya et al. (2017) sought to understand the influence of urban and rural lifestyles on the milk microbiota. The study used 15 profiles of the milk microbiota of urban women and 15 profiles of the milk microbiota of rural women living in tribal villages in India to establish the microbiome difference between rural and urban mothers and its effect on neonates. The study linked urbanization with altering the milk microbiota, a primary source of nutrition for newborns. Vaidya et al. (2017) found that urban and maternal microbial compositions are different. Rural mothers present a more diverse microbial community than urban mothers, with a p-value of < 0.001, which enhances microbiome maturation, making neonates less susceptible to infectious diseases [11]. 

Selma-Royo et al. (2020) explored the perinatal environment and its role in shaping microbiota colonization and a neonate's growth. The mode and place of delivery were identified as critical perinatal environmental factors responsible for shaping the microbiota of neonates with the potential to cause health consequences. The sequencing of the 16S rRNA gene was used to analyze the profile of the gut microbiota of 180 healthy infants at birth, after seven days, and after one month. After exposure to fecal supernatants from home births, the simulated intestinal epithelium demonstrated improved barrier function and maturation, as well as a stronger immunological response via TLR4 pathway activation and proinflammatory cytokine release, compared to infants delivered by cesarean section [8].

Kortman et al. (2023) compared the composition of mothers’ breast milk in tandem with the infant gut microbiota of mothers from distinct rural and urban regions in Vietnam. Even though no overall statistical significance was reached regarding the infant gut diversity between rural and urban areas, Kortman et al. (2023) found a strong association of geography with the microbial diversity of neonates. This observational study found that urban mothers were strongly associated with more potentially detrimental pathobionts than their rural counterparts [10].

Urban Ha Noi was found to have a higher abundance of pathobiontic taxa like Klebsiella and Citrobacter, but Bifidobacterium was less prevalent. Breast milk in rural Ha Long Bay had higher quantities of docosahexaenoic acid (DHA), eicosapentaenoic acid (EPA), selenium, and vitamin B12, compared to iron, zinc, and α-linolenic acid (ALA) in Ha Noi. The iron content of breast milk was positively associated with infant fecal Klebsiella and negatively associated with Bifidobacterium, while EPA and DHA contents were positively associated with Bifidobacterium [10].

Combellick et al. (2018) investigated the neonatal fecal microbiota of rural and urban areas or hospitals. The study reported postnatal microbiota differences in urban and rural mothers and babies. Hospitalization, as is the case for urban mothers, may affect the initial colonization that occurs during labor and birth, which can potentially persist in the infant microbiota [9].

Discussion

Even if there was no exposure in the womb, newborns are extensively bacterially colonized immediately after birth via their vaginal, fecal, and skin microbiota as well as via breast milk. This reaches the mesenteric lymph nodes before spreading throughout the body via systemic circulation [12,13]. At around one year of age, however, the microbiome of infants becomes increasingly complex and reaches a level similar to that of adults by the age of three [14].

This systematic review study made a comparison of rural and urban mothers to establish the underlying microbiome difference and its effect on neonates. The study results showed that urban or rural environments influence microbiome composition in different ways. Urban and rural environments affect the microbiome composition of mothers, albeit in different ways. Rural mothers are linked with high microbial diversity, immediate colonization of Bacteroides, and immediate immune responses when vaginal births are taken into account [8,15,16]. This leads to microbiome maturation, leading to improved metabolic development in newborns. This is contrary to urban mothers and babies who experience low microbial diversity and delayed colonization of Bacteroides. Microbial diversity restriction and bacteroid colonization delay among urban mothers and babies are linked with urban lifestyles and ways of doing things. For example, the caesarian section that is relatively high among urban mothers reduces the immune system and metabolic processes of newborns [17]. This is because the caesarian section has low amounts of Bacteroidetes in the initial stages after birth arising from low colonization of the body organs [18]. The caesarian section also influences breast milk composition. Breast milk is an important source of infant nutrition since it provides the protective nutrients infants require to proliferate during the first six months after birth [11]. 

The communication between the immune system and gut microbiota is intertwined; therefore, any changes in the microbial population can potentially disturb immunity, particularly maternal immunity. Maternal humoral immunity will protect the neonate first six months [19-21].

Other factors, such as the use of medication, the hospital environment, and diet, have been shown to alter the microbiome composition of urban mothers, affecting the development of newborns in early life. Medications, especially antibiotics used by urban mothers on a relatively large scale, are responsible for dysbiosis, which makes newborns susceptible to diseases such as sepsis [1]. In addition to handling treatment and feeding, the hospital environment enhances microbial transmission to neonates [9]. Diets such as fortified energy-protein supplementation lead to the depletion of microbial pathways and the phosphotransferase system [10]. The phosphotransferase system is critical for the uptake of carbohydrates by bacteria. Microbial contents vary and depend on their diet, lifestyles, and urban pollutants due to sanitary standards. Moreover, maternal traits such as birth methods, diet, and antibiotic use influence the maternal microbiome, which can result in reasons why rural moms differ from urban mothers. A study by Vlasova et al. showed that patients with neonate sepsis whose parents were living in polluted cities had considerably lower immunoglobulin levels than neonates from rural areas [6]. Antibiotics enter the environment through the excretions of humans and animals, through improper disposal and/or handling of unused medicines, and through direct environmental contamination in aquaculture or plant production. In the environment, the concentration of antibiotics was low, which can favor the acquisition of resistance [22-24].

Deng et al. (2023) in a randomized controlled trial study found that fortified energy-protein supplementation, regardless of whether it was balanced, increased microbiome diversity in urban expectant mothers. The fortified energy-protein supplementation was responsible for the depletion of microbial pathways and the phosphotransferase system. The phosphotransferase system is critical for the uptake of carbohydrates by bacteria. This study's results were simply explained because it considered sanitation and socioeconomic issues [25]. Lehtimäki et al. discovered a possible correlation between the microbiota of urbanized infants and their heightened susceptibility to asthma and allergic conditions. In contrast, rural aerobiomes were found to stimulate a T-regulatory and Th1-type immune response as opposed to an allergy- or asthma-specific Th2 response [26].

Breast milk contains beneficial bacteria like Bifidobacterium and Lactobacillus, which are essential for infant health. It also includes a variety of oligosaccharides that support the growth of bifidobacteria, primarily oligosaccharides that act as natural prebiotics [27]. Workforce obligations across urbanization gradients significantly contribute to breastfeeding cessation. Maternal intake of carbohydrates and proteins was linked to Prevotella-enriched clusters, while fiber, vegetable, protein, and polyphenol intake was linked to Ruminococcus-enriched clusters. These differences impacted the development of the infant's gut microbiota, potentially influencing later-life BMI. Studies in adults have shown that urban diets, sanitation, and antibiotics play a role in microbiota insufficiency syndrome [28].

The maternal gut microbiota has an indirect effect on fetal brain development through the production of vitamins and bacterial metabolites. The effects of the microbiota on the brain are evident within 12-14 hours of birth and include changes in cell death, microglial cell number and physiology, and cytokine expression. Dysbiosis of this gut-brain axis through urbanization can cause autism, attention deficits, and hyperactivity disorders [29].

In our hospital, most of the mothers are from rural areas. We observed less necrotizing enterocolitis, and despite sepsis, the activity of the babies was good, and they responded well to antibiotics, which drove us to do this review. We found evidence in favor of a rural microbiome in the prevention of sepsis, necrotizing enterocolitis, and long-term sequelae. However, there is a great need for more evidence in the form of more multicenter randomized trials. However, in adults, there is sufficient evidence for the long-term effects of the microbiome. One significant drawback of the research is, in a few cases, the lack of information regarding whether hospitalized lower cesarean section cases belonged to urban or rural regions. Our systematic review concentrated solely on urban and rural areas without taking into account other confounding variables.

## Conclusions

The study showed a microbiome difference in rural and urban mothers. The study identified microbiota differences linked to delayed maturation and neonatal infection risk. Urban mothers presented low microbial diversity and delayed colonization of Bacteroides compared to rural mothers. The effect of low microbial diversity and delayed colonization of Bacteroides is that newborns develop microbial taxa that cannot enhance neonates' immune systems, making them susceptible to infectious diseases. This also delays the maturation of organs in infants, slowing their development.

## References

[1] Brushett S, Sinha T, Reijneveld SA, de Kroon ML, Zhernakova A (2020). The effects of urbanization on the infant gut microbiota and health outcomes. Front Pediatr.

[2] Amir M, Brown JA, Rager SL, Sanidad KZ, Ananthanarayanan A, Zeng MY (2020). Maternal microbiome and infections in pregnancy. Microorganisms.

[3] Muglia LJ, Benhalima K, Tong S, Ozanne S (2022). Maternal factors during pregnancy influencing maternal, fetal, and childhood outcomes. BMC Med.

[4] Yao Y, Cai X, Ye Y, Wang F, Chen F, Zheng C (2021). The role of microbiota in infant health: from early life to adulthood. Front Immunol.

[5] Chong CY, Bloomfield FH, O'Sullivan JM (2018). Factors affecting gastrointestinal microbiome development in neonates. Nutrients.

[6] Vlasova OV, Hrytsiuk MI (2020). Effect of environmental pollution on indicators of humoral immunity in patients with neonatal sepsis. J Educ Health Sport.

[7] Morandini F, Perez K, Brot L (2023). Urbanization associates with restricted gut microbiome diversity and delayed maturation in infants. iScience.

[8] Selma-Royo M, Calatayud Arroyo M, García-Mantrana I, Parra-Llorca A, Escuriet R, Martínez-Costa C, Collado MC (2020). Perinatal environment shapes microbiota colonization and infant growth: impact on host response and intestinal function. Microbiome.

[9] Combellick JL, Shin H, Shin D (2018). Differences in the fecal microbiota of neonates born at home or in the hospital. Sci Rep.

[10] Kortman GA, Timmerman HM, Schaafsma A (2023). Mothers' breast milk composition and their respective infant's gut microbiota differ between five distinct rural and urban regions in Vietnam. Nutrients.

[11] Vaidya YH, Patel SH, Patel RJ, Pandit RJ, Joshi CG, Kunjadia AP (2017). Human milk microbiome in urban and rural populations of India. Meta Gene.

[12] Coscia A, Bardanzellu F, Caboni E, Fanos V, Peroni DG (2021). When a neonate is born, so is a microbiota. Life (Basel).

[13] Suárez-Martínez C, Santaella-Pascual M, Yagüe-Guirao G, Martínez-Graciá C (2023). Infant gut microbiota colonization: influence of prenatal and postnatal factors, focusing on diet. Front Microbiol.

[14] Fouhy F, Ross RP, Fitzgerald GF, Stanton C, Cotter PD (2012). Composition of the early intestinal microbiota: knowledge, knowledge gaps and the use of high-throughput sequencing to address these gaps. Gut Microbes.

[15] Kalbermatter C, Fernandez Trigo N, Christensen S, Ganal-Vonarburg SC (2021). Maternal microbiota, early life colonization and breast milk drive immune development in the newborn. Front Immunol.

[16] Zheng D, Liwinski T, Elinav E (2020). Interaction between microbiota and immunity in health and disease. Cell Res.

[17] Shaterian N, Abdi F, Ghavidel N, Alidost F (2021). Role of cesarean section in the development of neonatal gut microbiota: a systematic review. Open Med (Wars).

[18] Rutayisire E, Huang K, Liu Y, Tao F (2016). The mode of delivery affects the diversity and colonization pattern of the gut microbiota during the first year of infants' life: a systematic review. BMC Gastroenterol.

[19] Zhang H, Zhang Z, Liao Y, Zhang W, Tang D (2022). The complex link and disease between the gut microbiome and the immune system in infants. Front Cell Infect Microbiol.

[20] Conlon MA, Bird AR (2014). The impact of diet and lifestyle on gut microbiota and human health. Nutrients.

[21] Pickard JM, Zeng MY, Caruso R, Núñez G (2017). Gut microbiota: role in pathogen colonization, immune responses, and inflammatory disease. Immunol Rev.

[22] Cabello FC, Godfrey HP, Tomova A, Ivanova L, Dölz H, Millanao A, Buschmann AH (2013). Antimicrobial use in aquaculture re-examined: its relevance to antimicrobial resistance and to animal and human health. Environ Microbiol.

[23] Fick J, Söderström H, Lindberg RH, Phan C, Tysklind M, Larsson DG (2009). Contamination of surface, ground, and drinking water from pharmaceutical production. Environ Toxicol Chem.

[24] Gullberg E, Cao S, Berg OG, Ilbäck C, Sandegren L, Hughes D, Andersson DI (2011). Selection of resistant bacteria at very low antibiotic concentrations. PLoS Pathog.

[25] Deng L, Taelman S, Olm MR (2023). Effects of maternal fortified balanced energy-protein supplementation on the mother-infant gut microbiome: a sub-study of the MISAME-III randomized controlled trial (PREPRINT). medRxiv.

[26] Lehtimäki J, Thorsen J, Rasmussen MA (2021). Urbanized microbiota in infants, immune constitution, and later risk of atopic diseases. J Allergy Clin Immunol.

[27] Łubiech K, Twarużek M (2020). Lactobacillus bacteria in breast milk. Nutrients.

[28] Chen P, Billiar T (2020). Gut microbiota and multiple organ dysfunction syndrome (MODS). Adv Exp Med Biol.

[29] Kandpal M, Indari O, Baral B (2022). Dysbiosis of gut microbiota from the perspective of the gut-brain axis: role in the provocation of neurological disorders. Metabolites.

